# Bioinspired polydopamine nanoparticles as efficient antioxidative and anti-inflammatory enhancers against UV-induced skin damage

**DOI:** 10.1186/s12951-023-02107-7

**Published:** 2023-09-30

**Authors:** Jia Zhang, Yuqi Zhou, Zhaoting Jiang, Chenhui He, Bo Wang, Qi Wang, Zeqian Wang, Tong Wu, Xiaoqi Chen, Ziwei Deng, Chunying Li, Zhe Jian

**Affiliations:** 1 Department of Dermatology, Xijing Hospital Fourth Military Medical University, Xi’an, 710032 P. R. China; 2 Key Laboratory of Applied Surface and Colloid Chemistry, Ministry of Education, Shaanxi Key Laboratory for Advanced Energy Devices, Shaanxi Engineering Lab for Advanced Energy Technology, School of Materials Science and Engineering, Shaanxi Normal University, Xi’an, 710119 P. R. China

**Keywords:** Polydopamine, Nanoparticle, UV damage, Antioxidant, Inflammation, Sunscreen, Photoprotection

## Abstract

Excessive and prolonged ultraviolet radiation (UVR) exposure causes photodamage, photoaging, and photocarcinogenesis in human skin. Therefore, safe and effective sun protection is one of the most fundamental requirements. Living organisms tend to evolve various natural photoprotective mechanisms to avoid photodamage. Among them, melanin is the main functional component of the photoprotective system of human skin. Polydopamine (PDA) is synthesized as a mimic of natural melanin, however, its photoprotective efficiency and mechanism in protecting against skin damage and photoaging remain unclear. In this study, the novel sunscreen products based on melanin-inspired PDA nanoparticles (NPs) are rationally designed and prepared. We validate that PDA NPs sunscreen exhibits superior effects on photoprotection, which is achieved by the obstruction of epidermal hyperplasia, protection of the skin barrier, and resolution of inflammation. In addition, we find that PDA NPs are efficiently intake by keratinocytes, exhibiting robust ROS scavenging and DNA protection ability with minimal cytotoxicity. Intriguingly, PDA sunscreen has an influence on maintaining homeostasis of the dermis, displaying an anti-photoaging property. Taken together, the biocompatibility and full photoprotective properties of PDA sunscreen display superior performance to those of commercial sunscreen. This work provides new insights into the development of a melanin-mimicking material for sunscreens.

## Introduction

Human skin is the largest organ of the integumentary system, encompassing the outmost layer of the body. While skin serves as a shield to protect the internal body from the external environment, it is highly susceptible to damage caused by solar ultraviolet radiation (UVR) [1]. Currently, there is ample evidence articulating that excessive and constant UVR exposure causes erythema (sunburn), pigmentation (tanning), photocarcinogenesis, photoaging, and other adverse impacts on the skin. The UV spectrum (100–400 nm) is divided into three bands: ultraviolet A (UVA: 320–400 nm), ultraviolet B (UVB: 280–320 nm), and ultraviolet C (UVC: 100–280 nm). As absorbed by the ozone layer, virtually little UVC can reach the surface of the earth [2, 3]. UVB has high energy which contributes to direct epidermis damage, while UVA can penetrate straightly to the dermis of the skin, causing cumulative system disorder and destroying elastic and collagen fibers [4].

The utilization of sunscreen encompassed photoprotective compounds is feasible to shield the skin from UVR [5–8]. At present, a mixture of organic filters (avobenzone and octinoxate) and inorganic filters (Zinc Oxide and Titanium Dioxide) are extensively available in commercial UV sunscreens [9]. Nevertheless, these artificial UV filters encounter challenges including (1) they induce ROS generation and cellular DNA damage along with UVR after penetrating human skin [10–12], (2) their photodegradation property are both deleterious to ecosystems and human beings [10, 11]. Thereupon, it is imperative to develop a more effective sunscreen that not only follows new photoprotection concepts and mechanisms, more importantly, whose materials are biologically active and environmentally benign.

Skin pigmentation is the primary self-photoprotective mechanism in the human body to prevent skin damage caused by solar radiation. Melanin is a pigment produced by epidermal melanocytes, which plays a prominent role in building the primary self-photoprotective mechanism of human skin [13]. Melanin functions as a natural sunscreen, specialized in absorbing and dissipating UV radiation, as well as scavenging free radicals to minimize potential UV damage [14, 15]. Polydopamine (PDA) is synthesized as a melanin analog that shares similar physical and chemical properties with eumelanin (the main melanin with the ability to protect skin from photodamage) [16, 17]. Pioneer studies have validated that PDA nanoparticles (PDA NPs) have comparable light-protection properties with natural melanin. Besides, they possess simpler and controllable synthesis methods, making them a bio- and environment-friendly light-shielding agent [17]. In terms of functionality, PDA NPs can form a supranuclear cap in epidermal keratinocytes, mimicking the behavior of natural melanosomes, and therefore, providing an excellent UV-blocking function [14, 18]. Zhou et, al. [19] prepared polydopamine-grafted lignin (AL-PDA) as the sole active ingredient in a sunscreen, whose sun protection factor (SPF) value could reach 195.33 with a dosage ∼10 wt% lasting for over 8 h under UV radiation.

**Scheme 1:**
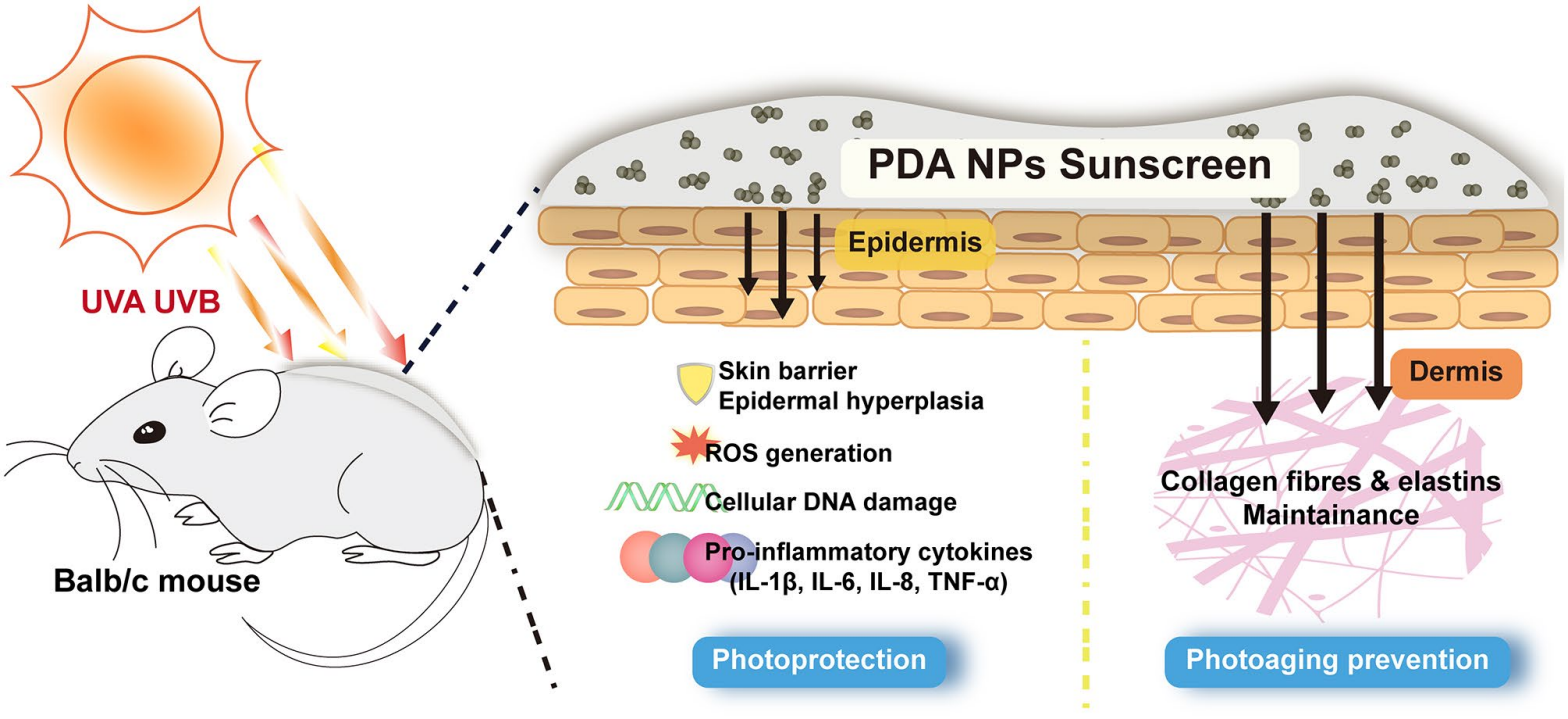
Illustration of PDA sunscreen in photoprotection and photoaging prevention

This study aims to introduce PDA NPs into commercial suncream to formulate a PDA sunscreen and investigate its performance in the improvement of photoprotection and photoaging prevention (Scheme 1). Based on the following results, we consider this PDA sunscreen with highly biomimetic photoprotective characterization may provide new insights into nanoparticle-introduced sunscreen manufacture.

## Materials and methods

### Synthesis of PDA

Polydopamine nanoparticles (PDA NPs) were prepared following a modified literature method. The specific process was described as follows: dopamine hydrochloride (2 g/L, Ruixibio, China) was dissolved in 30% aqueous ethanol, followed by adding ammonia solution (0.1 mol/L) and reacting at 37 ℃ for 24 h. PDA NPs were obtained by centrifugation (8000 rpm, 30 min), washed with deionized water, and freeze-dried overnight.

### Characterization of PDA NPs

The morphology of the freeze-dried PDA NPs samples was investigated using a transmission electron microscope (TEM, JEOL, JEM-F200, Japan) operated at 200 kV with fit magnification. The distribution of C, N, and O elements in samples was observed by elemental mappings. UV-vis absorption spectroscopy of the samples was detected by UV-vis spectrophotometer (Perkin-Elmer, Lambda650, USA).

### DPPH free-radical scavenging activity assay

The antioxidant activity of PDA NPs was evaluated by measuring their ability to scavenge 2,2-diphenyl-1-picrylhydrazyl (DPPH), purchased from Sigma-Aldrich (Cat# 257,621, MO, USA). 100 μL of each sample was added to a DPPH methanolic solution (900 μL, 0.1 mM) and incubated at room temperature for 30 min in the dark. Absorbance was measured at 517 nm against a blank using a spectrophotometer. The percentage of antioxidant activity was calculated by the following formula: DPPH scavenging (%) = [(A DPPH − A sample)/A DPPH] × 100.

### Cell culture and treatment

Human epidermal keratin-forming (HaCaT) cells were purchased from Procell Life Sciences Co., Ltd. (#Cat: CL-0090, Wuhan, China). The HaCaT cells were cultured in a MEM medium (#PM150410, Procell Life Sciences Co., Ltd., China) containing 15% Fetal Bovine Serum (FBS) (#164210-50, Procell Life Sciences Co., Ltd., China) and 1% Penicillin-Streptomycin Solution, maintained at 37 °C with 5% CO_2_. For UV irradiation, HaCaT cells were seeded in 6-well plates at a density of 2.0 × 10^5^ cells per well. After culturing overnight, different concentrations of PDA NPs were added and incubated for 24 h. Then, the cells were irradiated with an indicated dose of UV irradiation (Nanjing Huaqiang Electronics Co., LTD, China) after they were rinsed three times with PBS.

### Cell viability

We used cell counting kit-8 (CCK-8, Glpbio, CA, USA). assay and flow cytometry analysis to assess the potential cellular cytotoxicity of PDA NPs. HaCaT cells were seeded and cultured overnight, then incubated with different concentrations of PDA NPs for 24 h. In the CCK-8 assay, the cells were rinsed three times with PBS. Subsequently, 100 μL CCK-8 working solution (CCK-8: MEM = 1:10) was added to each well and incubated for 2 h. The optical density (OD) was measured at 450 nm using a microplate reader (Multiskan GO, ThermoScientific, America) and the cell viability was calculated as (OD test group – OD blank group) / (OD control group – blank group) × 100%. For flow cytometry analysis, HaCaT cells were stained with Fixable Viability Dye eFluor^™^ 780 (eBioscience) according to the manufacturer’s instructions.

### Preparation of sunscreen

For sunscreen applications, 1.25 g, 2.5 g, and 5 g PDA NPs were added to 50 g suncream configured as mass ratios of 2.5 wt%, 5 wt%, and 10 wt%, respectively.

### Animal experiments

Female BALB/c mice were purchased from the laboratory animal center of the Fourth Military Medical University (Xi’an, Shaanxi, China) and maintained in a specific pathogen-free mouse facility. After acclimatization for a week, mice were randomly assigned to three groups. For inducing photodamages, the UV irradiation scheme was at week 1: mice were irradiated with 2200 mJ/cm^2^ UVA and 110 mJ/cm^2^ UVB; at week 2: mice were irradiated with 4400 mJ/cm^2^ UVA and 220 mJ/cm^2^ UVB; at week 3–9: 8800 mJ/cm^2^ UVA and 440 mJ/cm^2^. Specifically, the tubes (Nanjing Huaqiang Electronics Co., LTD, China) for UVA and UVB radiation were set at 340 nm and 314 nm, respectively. The final power was detected by a UV illuminance meter (Cat# TM-213) manufactured by TENMARS (Taiwan, China). Meanwhile, the photodamaged skin of mice was applied with either (1) commercial sunscreen (vehicle group) or (2) 2.5 wt%, 5 wt%, and 10 wt% PDA NPs sunscreen respectively (PDA group) (refer to Fig. 2A). After 9 weeks, mice were sacrificed and their dorsal skin biopsies were fixed in 10% (v/v) formalin solution for histological analysis. All animal experiments were followed by ethical regulations approved by the Subcommittee on Research Animal Care of the Fourth Military Medical University (Xi’an, China).

### Clinical skin score

After the BALB/c mice were exposed to UV irradiation, the four-point Likert scale was used to assess the photodamaged symptoms, including erythema/hemorrhage, scarring/dryness, edema, and excoriation/ erosion. Each symptom was graded on a scale from 0 to 3 (none, 0; mild, 1; moderate, 2; severe, 3). The severity of skin damage was calculated by the sum of the individual scores, ranging from 0 to 12.

### Hematoxylin and eosin (H&E) staining

The fixed skin biopsies were embedded with paraffin and sectioned into 5 μm. The sections were deparaffinized and stained with hematoxylin and eosin. The histological changes were observed under a light microscope.

### Skin surface physiological analysis

In short, mice were shaved one day before the transepidermal water loss (TEWL) assessment. Under standard environmental conditions (21–22 ℃ and 50–55% humidity), mice were anesthetized and TEWL was measured via a multifunctional skin physiology monitor (MPA10, Courage-Khazaka Electronic GmbH, Cologne, Germany).

### Intracellular ROS measurement

ROS was detected using 2,7-dichlorodihydrofluorescein diacetate (DCFH-DA) or dihydroethidium (DHE). After 24 h of PDA NPs incubation and UV irradiation (UVA 200 mJ/cm^2^ + UVB 5 mJ/cm^2^ or UVA 400 mJ/cm^2^ + UVB 10 mJ/cm^2^), HaCaT cells were either stained with 10 μM DCFH-DA or 10 μM DHE in the dark for 30 min. The intensity of fluorescence was detected via Zeiss LSM880 confocal laser scanning microscope (Zeiss, German) or BD LSRFortessa™ Flow Cytometer (BD Biosciences, San Jose, CA, USA).

### Comet assay

Comet assay was performed following the manufacturer protocol (Trevigen, 4250-050-K). The stained nuclei were then examined by a Nikon Ti-S fluorescence microscope (Nikon, Tokyo, Japan), and then analyzed with CaspLab (CASP) software.

### Immunohistochemistry (IHC) and immunofluorescence (IF) analysis

Paraffin-embedded tissue sections were deparaffinized and antigen retrieved in sodium citrate buffer (10 mM, pH 6.0). After the sections were blocked by goat serum, primary antibodies were added and incubated overnight: anti-tumor protein (p53) antibody (#ab32389, Abcam, 1: 100), anti-filaggrin (FLG) antibody (#ab81468, Abcam, 1: 200), anti-MPO antibody (#ab208670, Abcam, 1: 100), anti-MMP-1 antibody (#ab134184, Abcam, 1: 200), anti-MMP-3 antibody (#ab52915, Abcam, 1: 200). For IHC staining, the sections were stained with the corresponding secondary antibodies, visualized with diaminobenzidine in the Envision System, counterstained with Mayer’s hematoxylin, and mounted with a non-aqueous mounting medium. For immunofluorescence staining, secondary antibodies Cy3 (Zhuangzhibio, Xi’an, China) were used according to the sources of primary antibodies. Meanwhile, cell nuclei were labeled with Hoechst 33,258 (1:1000, cat. C1011, Beyotime, Shanghai, China). Immunofluorescent images were viewed by Zeiss LSM880 confocal laser scanning microscope (Zeiss, German).

### Western blot (WB) analysis

The western blot technique was used to detect the protein expression of HaCaT cells. At the end of the treatment, the cells were washed with PBS 3 times and lysed with RIPA buffer for 30 min. The concentration of protein was determined by the BCA Protein Assay Kit (Elabscience, Wuhan, China). The protein samples were subjected to SDS-PAGE gel electrophoresis and then transferred to PVDF membranes. The blots were incubated overnight at 4 ℃ with the following primary antibodies: GADPH (ab8245, Abcam, 1: 5000), p53 (ab32389, Abcam, 1: 5000), MPO (ab208670, Abcam, 1: 1000), TNF-α (ab183218, Abcam, 1: 2000), IL-1β (ab254360, Abcam, 1: 1000), MMP-1 (ab134184, Abcam, 1: 1000) and MMP-3 (ab52915, Abcam, 1: 5000). The membranes were visualized using a chemiluminescence analyzer (Bio-Rad, Hercules, CA, USA). The relative density of protein bands was analyzed using the ImageJ software, and optical density values were normalized to GADPH.

### Quantificational real-time PCR (qRT-PCR) analysis

For qRT-PCR analysis, cells RNA was isolated according to the purification of RNA using Trizol (TRI reagent) and reversed transcribed into cDNA via Superscript first strand synthesis kit (Invitrogen) in the manufacturer’s protocol. The qRT-PCR was performed using the PrimeScript^™^ RT Master Mix kit (Cat# RR036A, TaKaRa, Tokyo, Japan). All primers were synthesized by TsingKe Biological Technology (Beijing, China). The sequences of PCR primers are listed in Tables 1 and 2. qRT-PCR was performed in the following conditions: 95 ℃ for 3 min; 40 cycles: 95 ℃ for 15 s, 60 ℃ for 15 s, 72 ℃ for 15 s; then for melting curve analysis. The 2 − ΔΔCT method was used to quantify the relative expression of these genes.

**Table 1:** (Specific primers used in qRT-PCR analysis.)

Gene	Primer	Sequence (5’→3’)
Human β-actin	F	GGCTACAGCTTCACCACCAC
	R	TGCGCTCAGGAGGAGC
Human TNF-а	F	CTCTTCTGCCTGCTGCACTTTG
	R	ATGGGCTACAGGCTTGTCACTC
Human IL-1β	F	TTCGACACATGGGATAACGAGG
	R	TTTTTGCTGTGAGTCCCGGAG
Human IL-6	F	AGACAGCCACTCACCTCTTCAG
	R	TTCTGCCAGTGCCTCTTTGCTG
Human IL-8	F	ACTGAGAGTGATTGAGAGTGGAC
	R	AACCCTCTGCACCCAGTTTTC

**Table 2:** (Specific primers used in qRT-PCR analysis.)

Gene	Primer	Sequence (5’→3’)
Mouse β-actin	F	GGCTGTATTCCCCTCCATCG
	R	CCAGTTGGTAACAATGCCATGT
Mouse MMP-1	F	GGGGCTTTGATGTACCCTAGC
	R	TGTCACACGCTTTTGGGGTTT
Mouse MMP-3	F	CGGTTCCGCCTGTCTCAAG
	R	CGCCAAAAGTGCCTGTCTT

### Enzyme-linked immunosorbent assay (ELISA)

IL-6 and IL-8 in the supernatants of HaCaT cells were determined via ELISA kits (IL-6, Cat# E-EL-H6156; IL-8, Cat# E-EL-H6008; Elabscience; China) followed by the corresponding manufacturer’s instruction.

### Masson’s trichrome stain

The sections were deparaffinized in water and then fixed in Bouin’s solution, stained with celestine blue for 2–3 min. Afterward, the sections were washed and stained with Mayer’s hematoxylin solution for 2–3 min, followed by differentiated with 1% ethanol hydrochloride for a few seconds and rinsed with water for 10 min. Subsequently, we stained sections with the Li chunhong acid fuchsin staining solution for 10 min and rinsed them with distilled water. Then, the sections were treated with a phosphomolybdic acid solution for approximately 10 min and decanted. Thereafter, the sections were stained with an aniline blue staining solution for 5 min without washing. At last, the sections were treated with 0.1–0.3% glacial acetic acid solution for 2 min, followed by dehydration, and the slices were mounted.

### Statistical analysis

All results are presented as the mean ± standard deviation (SD). Data were analyzed by one-way ANOVA and Bonferroni multiple comparison test. All experiments were performed in triplicate and *p* < 0.05 was considered statistically significant. All statistical analyses were performed using the GraphPad Prism 8.0 version (GraphPad Software Inc., San Diego, CA, USA) based on the distribution of data.

## Results

### Characterization and biocompatibility of PDA NPs

**Fig. 1:**
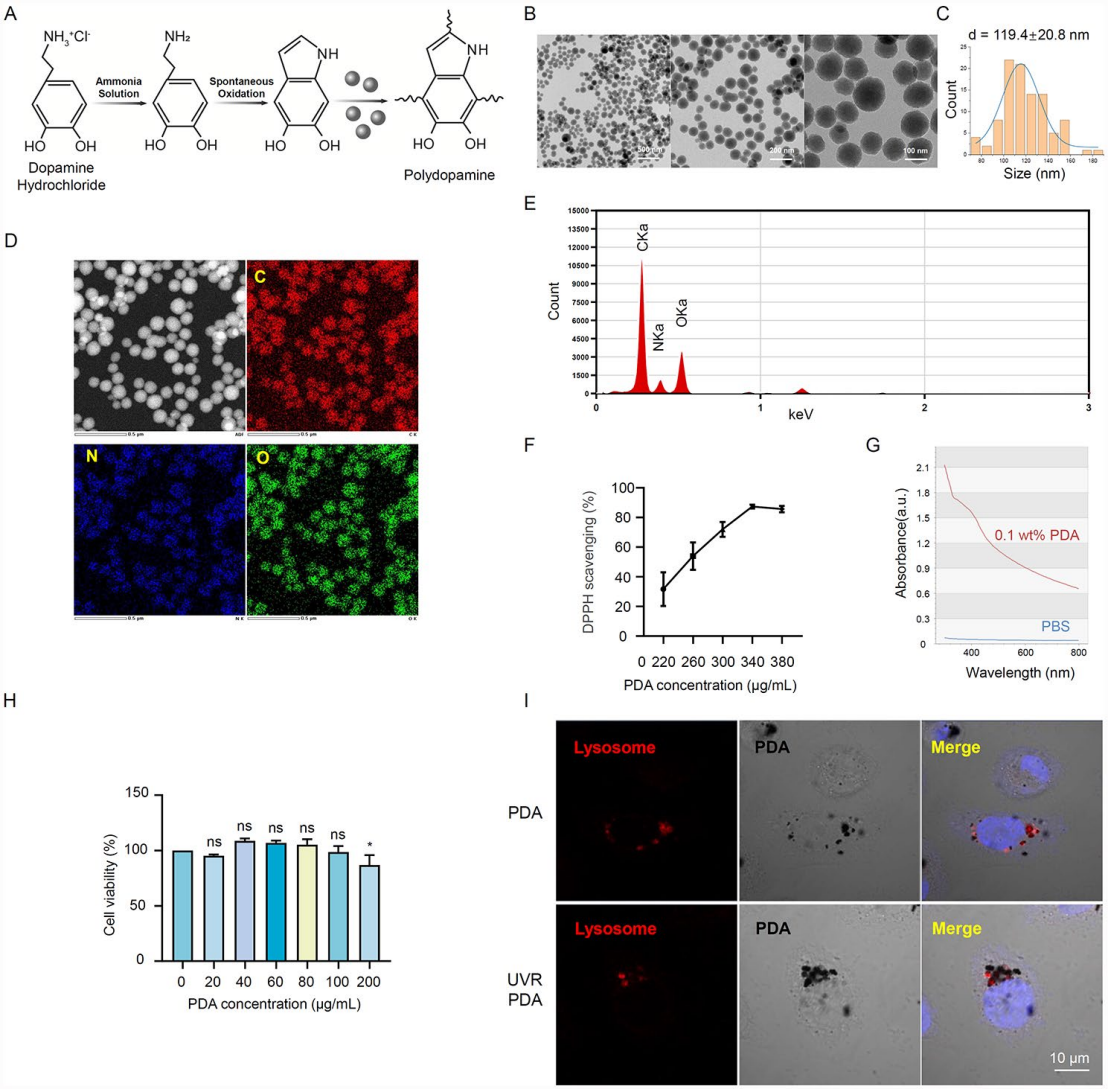
Characterization and biocompatibility of PDA NPs. (**A**) The schematic diagram illustrates the synthesis of PDA nanoparticles (PDA NPs). PDA NPs were prepared via oxidized and self-polymerized dopamine hydrochloride in an alkaline water-ethanol solution. (**B**) Transmission electron microscopy (TEM) images of PDA NPs. (**C**) Quantitative analysis of particle size distribution of PDA NPs (n = 100). (**D, E**) Elemental mapping and energy dispersive spectra of PDA NPs. Scale bar = 500 nm. (**F**) DPPH free-radical scavenging ability of different concentrations of PDA NPs (0, 220, 260, 300, 340, and 380 μg/mL). (**G**) UV-absorbance spectra of 0.1 wt% PDA and PBS solution. (**H**) HaCaT cells (an immortal cell line of human keratinocytes) were incubated with different concentrations of PDA NPs (0, 20, 40, 60, 80, 100, and 200 μg/mL) for 2 h. The cell viability of HaCaT cells was detected by CCK-8 assay. Scale bar = 10 μm. (**I**) Confocal laser scanning microscopy images of PDA NPs intake by HaCaT cells and colocalized with lysosomes (UVR-/+). Lysosomes were stained with LysoTracker Red DND-99 (red). Nuclei were stained with Hoechst 33,258 (blue); PDA NPs were black in HaCaT cells under a bright field. Data represent the mean ± SD. *p < 0.05.

As shown in Fig. 1A, PDA NPs were prepared via oxidized and self-polymerized dopamine hydrochloride in an alkaline water-ethanol solution at room temperature. The synthesized PDA NPs exhibited a monodisperse spherical structure and dispersed in an average diameter of 119.4 nm (Fig. 1B and C). The elemental mapping images showed that the C, N, and O elements were uniformly distributed within the spherical-shaped PDA NPs (Fig. 1D, E). To investigate the antioxidant capacity of PDA NPs in vitro, 2,2-diphenyl-1-picrylhydrazyl (DPPH) free radical scavenging assay showed that PDA NPs could scavenge DPPH free radicals in a dose-dependent manner, whose scavenging rate reaches 89.6% at a concentration of 380 μg/mL (Fig. 1F). Additionally, 0.1 wt% PDA NPs exhibited an excellent absorbance ability towards 280 nm-800 nm compared with the PBS solution, which was detected by UV absorption spectrophotometry (Fig. 1G).

Biocompatibility is the main criterion for utilizing biomaterials in clinical applications [20], therefore, we initially examined the cytotoxicity of PDA NPs. As shown in Fig. 1H, the cell viability of HaCaT cells (an immortal cell line of human keratinocytes) still retained at 80% after incubation with 200 μg/mL PDA NPs, which mirrored that PDA NPs have a marginal influence on cell viability. Given this, we selected concentrations within 200 μg/mL of PDA NPs in further cellular experiments. To observe the cellular uptake of PDA NPs, confocal laser scanning microscopy (CLSM) confirmed that PDA NPs were co-localized with the lysosomes surrounding nuclear in HaCaT cells after incubation of 48 h (Fig. 1I), consistent with the observation reported by Huang et, al. [17]. The hypothesis is PDA NPs might deliver to lysosomes and subsequent accumulation surrounding nuclear to form an artificial cap. This lysosome-mediated degradation and accumulation of PDA NPs vividly mimic natural melanosomes. Altogether, we successfully synthesized PDA NPs that possess spherical structures, antioxidant capacity, and UV absorption ability. In particular, PDA NPs were biocompatible and could be metabolized well out of the cell, thus they could be a potential candidate for biological applications.

### Photoprotection of PDA NPs on UV-induced skin damage in Balb/c mice

**Fig. 2:**
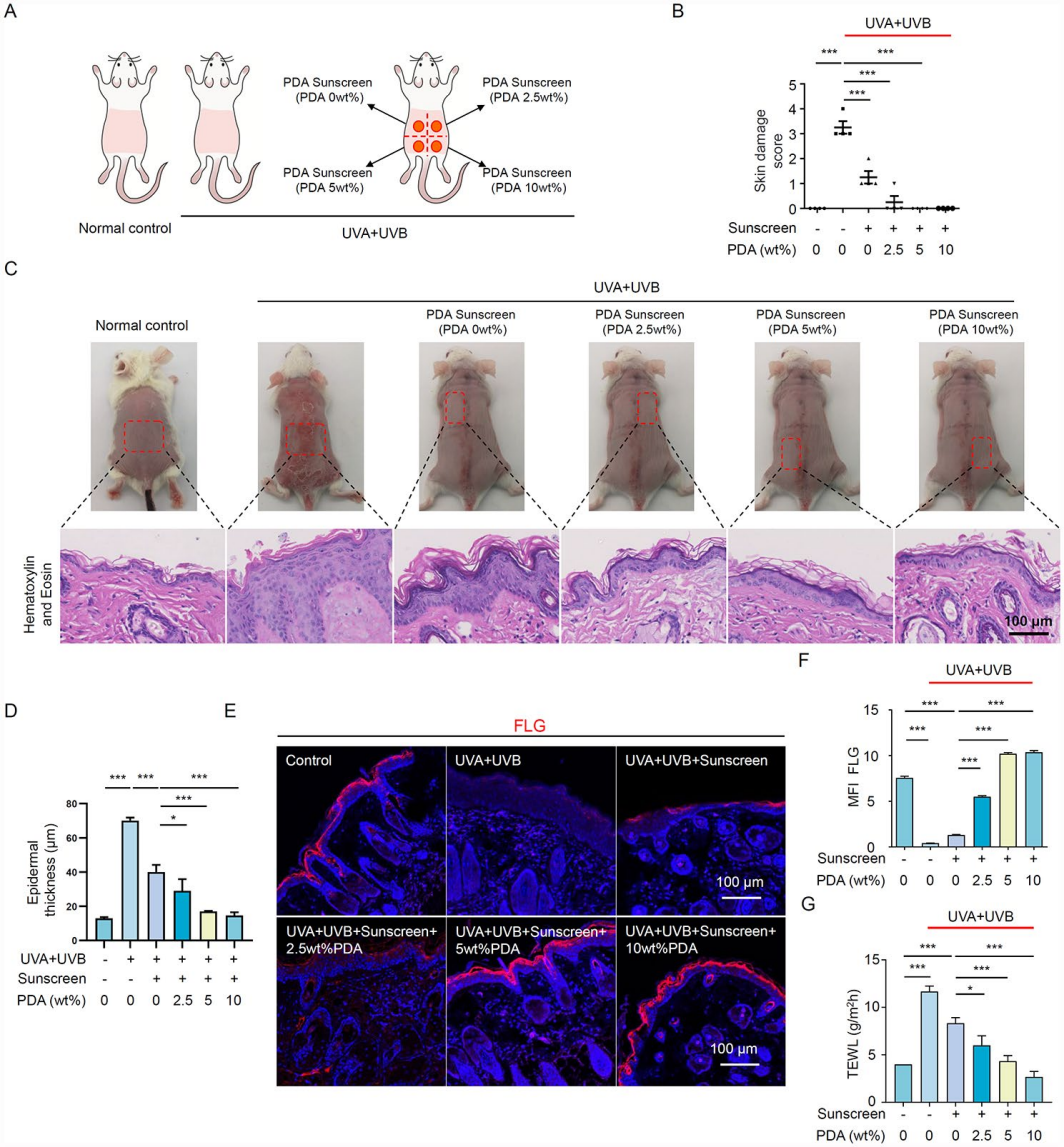
Photoprotection of PDA NPs sunscreen on UV-induced skin damage of Balb/c mice. (**A**) Schematic diagram of the commercial sunscreen or PDA NPs sunscreen applied topically in the dorsal skin of Balb/c mice. Mice were exposed to UVR (UVA + UVB) for 9 weeks. Meanwhile, several randomly selected UVR skin-damaged mice were treated with either (1) commercial sunscreen (vehicle group) or (2) prepared with different concentrations of PDA NPs (2.5 wt%, 5 wt%, and 10 wt%, PDA sunscreen group). (**B**) Quantifications of the skin severity scores of dorsal skin in Balb/c mice after experimental treatments. The results were calculated by the sum of scores in each term referred to four-point Likert scale, including the symptoms of erythema/hemorrhage, edema, scaling/dryness, and excoriation/erosion. (**C**) Representative photographic and H&E staining images of Balb/c mice in each group. (**D**) Quantitative analysis of epidermal thickness measured in H&E staining. (**E, F**) Representative immunofluorescent images and quantification of filaggrin (FLG) signal in the dorsal skin of Balb/c mice after different experimental treatments. FLG were stained with Cy3 and nuclei were stained with Hoechst 33,258 (blue). (**H**) Quantification of transepidermal water loss (TEWL) evaluated by Tewameter with colorimeter MPA-580(Courage & Khazaka, Cologne, Germany). N = 4 in each group. All data represent the mean ± SD. *p < 0.05, **p < 0.01 and ***p < 0.001.

The above results demonstrated that synthetic PDA NPs exhibit UV absorption ability and biocompatibility. Here, we mixed PDA NPs into commercial sunscreen and testified whether PDA-based sunscreen efficiently protects skin from UV-induced damage. Figure 2 A illustrated the schematic diagram of PDA sunscreen application on UV photodamaged Balb/c mice. After 9 weeks of application, the dorsal skin of mice was photographed and removed for histological analysis. To our knowledge, UVB (280–320 nm) is the leading trigger of acute damage to the skin, which can be evidenced by the occurrence of erythema, edema, pigmentation, crust, and immunity imbalance [21]. Thus, we utilized the four-point Likert scale to evaluate the photodamage-related symptoms of mice, including erythema/hemorrhage, edema, scaling/dryness, and excoriation/erosion. Scoring showed that the dorsal skin of mice was remarkably aberrant after UVR, meanwhile, the skin in the vehicle group presented moderate injury symptoms (Fig. 2B). Notably, the appearance of skin in the PDA sunscreen group resembled healthy skin, whose erythema score was lowest when PDA concentration reach 10 wt% (Fig. 2B).

Next, H&E staining demonstrated that there was a > 4-fold increase (68.3 ~ 71.8 μm) in epidermal thickness and inflammatory cells infiltrating the skin after UVR (Fig. 2C, D). Compared to the normal control (12.2–13.7 μm), the epidermal thickness in the vehicle group was 36.1 to 44.4 μm, whereas the PDA sunscreen shrank the epidermal hyperplasia most prominently (13.2–16.7 μm) (Fig. 2C, D). Skin barrier Filaggrin (FLG) is the key protein that maintains skin barrier function [22]. Herein, the immunofluorescence staining revealed that UVR dramatically disrupted the expression of FLG in the epidermis, which only could be slightly rescued by commercial sunscreen (Fig. 2E, F). Instead, PDA sunscreen prompted the expression of FLG in the epidermis in concentrations of 5 wt% and 10 wt% (Fig. 2E, F), suggesting the superior skin barrier protection ability of PDA sunscreen. Beyond that, we utilized a Tewameter device to evaluate the performance of PDA sunscreen in water preservation of the skin. In agreement with the above results, PDA sunscreen reduced the transepidermal water loss (TEWL) robustly after UVR (Fig. 2G). Collectively, our results suggest that PDA sunscreen could maintain the resilience of the skin to UV stress. Notably, we prepared a concentration of up to 10 wt% PDA in sunscreen, however, 5 wt% PDA sunscreen has already shown a satisfactory protective effect, as well as avoiding the color of sunscreen being too dark to utilize aesthetically.

### PDA NPs ameliorate UVR-induced cell death and scavenge UVR-induced ROS in keratinocytes

**Fig. 3:**
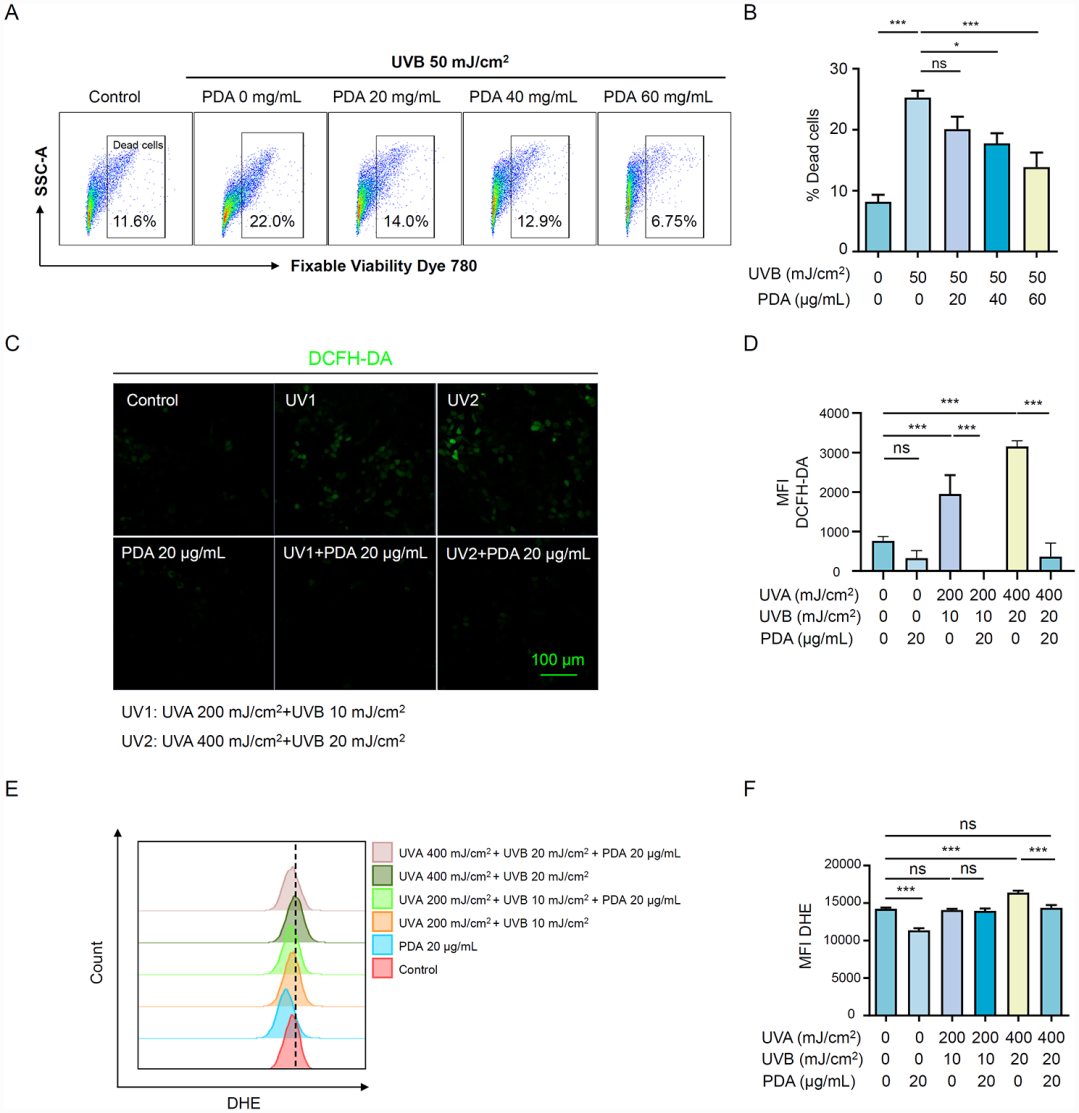
PDA NPs ameliorate UVR-induced Cell Death and Scavenge UVR-induced ROS in keratinocytes. (**A, B**) Flow cytometry analysis plots and quantifications of cell death of HaCaT cells pretreated with PDA NPs (0, 20,40, 60 mg/mL) for 24 h followed by UV irradiation (UVB 50 mJ/cm^2^). (**C, D**) Representative fluorescent images and quantification of ROS level in HaCaT cells pretreated with 20 mg/mL PDA NPs for 24 h followed by UV irradiation (UVA 200 mJ/cm^2^ + UVB 10 mJ/cm^2^, or UVA 400 mJ/cm^2^ + UVB 20 mJ/cm^2^). The ROS level was probed by DCFH-DA (green). Scale bar = 100 μm. (**E, F**) Flow cytometry analysis plots and quantifications of ROS level in HaCaT cells pretreated with 20 mg/mL PDA NPs for 24 h followed by UV irradiation (UVA 200 mJ/cm^2^ + UVB 10 mJ/cm^2^, or UVA 400 mJ/cm^2^ + UVB 20 mJ/cm^2^). The ROS level was detected by DHE fluorescent probes (PE-TexasRed). All data represent the mean ± SD. *p < 0.05, **p < 0.01 and ***p < 0.001

Previously, we found that UVR steered PDA NPs to aggregate in lysosomes compared to the normal condition (Fig. 1I), which suggests that UVR may accelerate the utilization and degradation process of PDA NPs in HaCaT cells. From this, we wondered whether PDA NPs could protect keratinocytes under UVR. HaCaT cells were pre-treated with different concentrations of PDA NPs (0, 20, 40, and 60 μg/mL) for 24 h, and then exposed to UVB at 50 mJ /cm^2^. Flow cytometry analysis revealed that UVR caused ~ 22% HaCaT cell death, whereas PDA NPs pre-treatment ameliorated cell death in a concentration-dependent manner (Fig. 3A, B). Evidence suggested that UV irradiation induces reactive oxygen species (ROS) accumulation, which interacted with various intracellular components to cause a malfunction in cells [23, 24]. Given this, we further investigated the effectiveness of PDA NPs in hampering UV-induced ROS generation. After exposed to UVR (UVA 200 mJ/cm^2^ + UVB 10 mJ/cm^2^, or UVA 400 mJ/cm^2^ + UVB 20 mJ/cm^2^) for generating ROS overproduction in HaCaT cells, we used the fluorescent probes of ROS 2,7-dichlorofluorescein diacetate (DCFH-DA) and dihydroethidium (DHE) to quantify ROS level. As illustrated by CLSM and flow cytometry analysis, PDA NPs (20 μg/mL) pre-treatment could prominently reduce intracellular ROS generation of HaCaT cells, whose ROS level lowered close to the normal control group (Fig. 3C-F). Altogether, our results revealed that PDA NPs exhibited potent protection and ROS neutralization of keratinocytes confronting UV irradiation.

### PDA NPs reduced UV-induced DNA damage

**Fig. 4:**
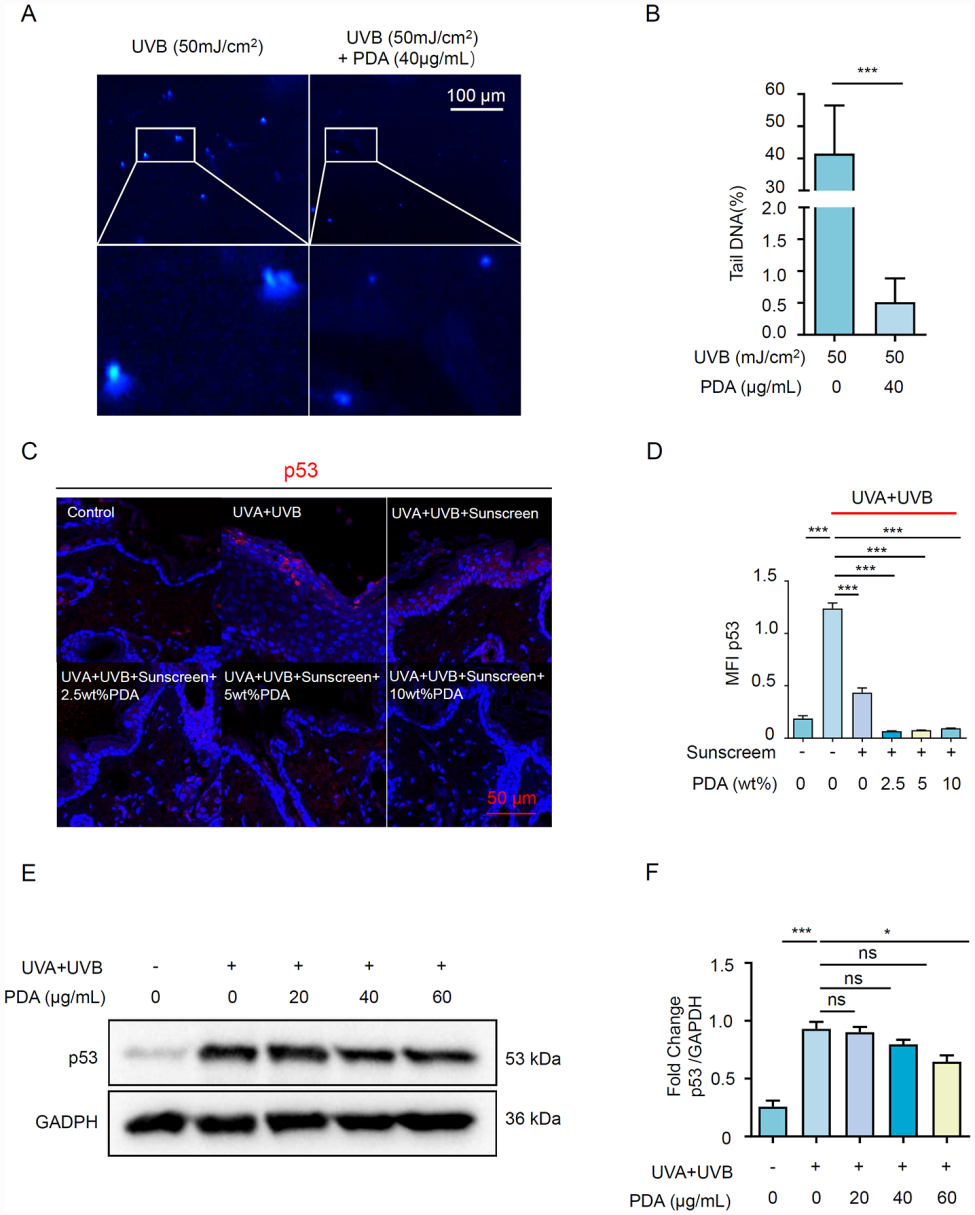
PDA NPs reduce UV-induced DNA damage. (**A, B**) Representative immunofluorescent images of the nuclei in HaCaT cells pretreated with 40 mg/mL PDA NPs for 24 h followed by 50 mJ/cm^2^ UVB. The comet tail of damaged DNA was stained with a Comet assay kit from the R&D system. The length of the comet tail was analyzed by CaspLab (CASP) software. (**C, D**) Representative immunofluorescent images and quantification of p53 signal in the dorsal skin of Balb/c mice after different experimental treatments (n = 4). P53 was stained with Cy3 (red) and nuclei were stained with Hoechst 33,258 (blue). (**E**) The p53 protein level of HaCaT cells was detected after being pretreated with different concentrations of PDA NPs (0, 20, 40, and 60 mg/mL) for 24 h, followed by exposure to UVR. (**F**) Semi-quantitative analysis of p53 was normalized to GAPDH. All data represent the mean ± SD. *p < 0.05, **p < 0.01 and ***p < 0.001

The acute UV irritation induced the accumulation of DNA damage, leading to mutations and potential photocarcinogenesis [25]. To examine the DNA protection ability of PDA NPs, we performed a comet assay analysis in HaCaT cells, which were pre-treated with PDA NPs and followed by UVR exposure. After UVR exposure, we found DNA moves away from nuclei of HaCaT cells and a large amount of comet tail appeared, which implied the occurrence of cellular DNA damage. Instead, PDA NPs pre-treatment drastically lessens the length of comets (Fig. 4A, B). P53 is known as the “guardian of the genome” since p53 protects the integrity of DNA [26], therefore we validated p53 expression *in vivo and in vitro*. Immunofluorescence staining revealed that PDA NPs significantly reduced p53 expression in UVR-exposure lesions of mice (Fig. 4C, D). Likewise, UVR-induced p53 expression in HaCaT cells was down-regulated after incubation of 60 μg/mL of PDA NPs (Fig. 4E, F).

### PDA NPs relieve UVR-induced inflammation

**Fig. 5:**
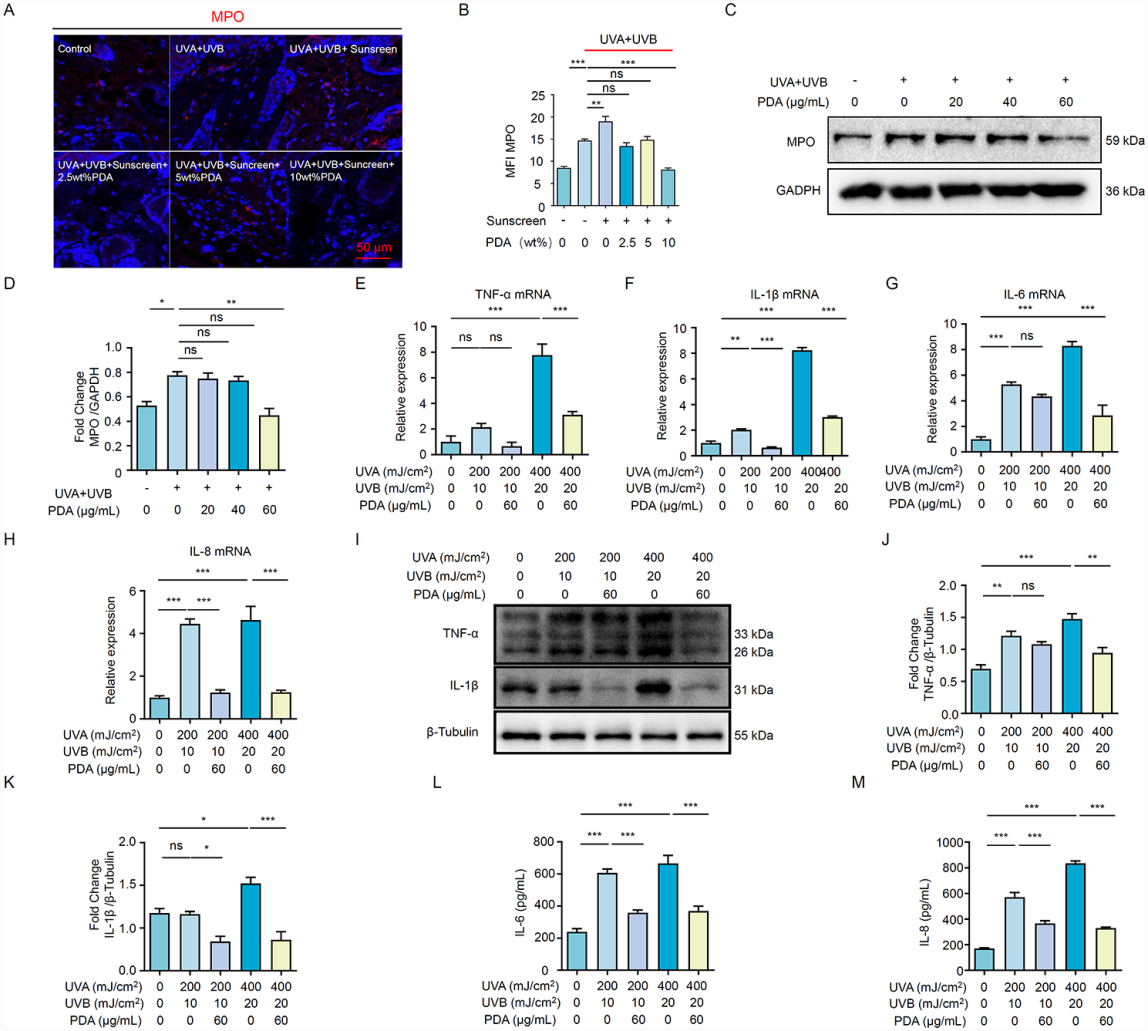
PDA NPs relieve UVR-induced inflammation. (**A, B**) Representative immunofluorescent images and quantification of MPO signal in the dorsal skin of Balb/c mice after different experimental treatments (n = 4). MPO was stained with Cy3 (red) and nuclei were stained with Hoechst 33,258 (blue). (**C**) The MPO protein level of HaCaT cells was detected after being pretreated with different concentrations of PDA NPs (0, 20, 40, and 60 mg/mL) for 24 h, followed by exposure to UVR. (**D**) Semi-quantitative analysis of p53 was normalized to GAPDH. (**E-H**) The mRNA expression levels of TNF-α, IL-1β, IL-,6 and IL-8 of HaCaT cells were detected by qRT-PCR after being pretreated with different concentrations of 60 mg/mL PDA NPs for 24 h, followed by exposure to UVR. The mRNA expression levels of these genes were normalized to β-actin. (**I**) The expression levels of TNF-α and IL-1β or β-Tubulin (as an internal control) were determined by western blotting. (**J, K**) The TNF-α and IL-1β protein level was detected in the lysates extracted from skin biopsies of Balb/c mice in different groups and quantified via normalization to β-Tubulin (n = 3). (**L, M**) The secretion of IL-6 and IL-8 in supernatants of HaCaT cells after indicated treatments was detected by their ELISA kit. All data represent the mean ± SD. *p < 0.05, **p < 0.01 and ***p < 0.001

The overproduction of ROS provokes an imbalance of pro-oxidant species, not only leading to oxidative damage in cellular macromolecules (e.g. DNA, proteins, and lipids), but also invokes the occurrence of inflammation [27, 28]. To determine whether PDA pre-treatment could ameliorate UVR-induced inflammation, first, we investigated the expression of myeloperoxidase (MPO), a surrogate for assessing the number of infiltrated neutrophils after UV irradiation. Immunofluorescence staining revealed that MPO expression was down-regulated in the UVR-induced lesions of 10%wt PDA sunscreen (Fig. 5A-B). In vitro, the expression of MPO in HaCat cells was elevated after UV radiation, whereas it was diminished by pre-treatment of 60 μg/mL PDA NPs. (Fig. 5C-D). Epidermal keratinocytes play a pronounced role in secreting UV-damaged proinflammatory factors, resulting in skin edema and an influx of neutrophils [29]. Herein, we further examined major inflammatory cytokines that play a central role in the onset of photodamage. Quantificational real-time polymerase chain reaction (qRT-PCR) validated that PDA NPs reduced the mRNA levels of TNF-α, IL-1β, IL-6, and IL-8 of HaCaT cells after UVR (Fig. 5E-H). In agreement with the above results, western blot analysis and enzyme-linked immunosorbent assay (ELISA) demonstrated that TNF-α, IL-1β, IL-6, and IL-8 expression or secretion were significantly prohibited by PDA NPs (Fig. 5I-M). Taken together, our results indicated a promising effect of PDA NPs on easing the inflammation of keratinocytes under UVR.

### PDA NPs suppress UV-Induced collagen degradation and photoaging

**Fig. 6:**
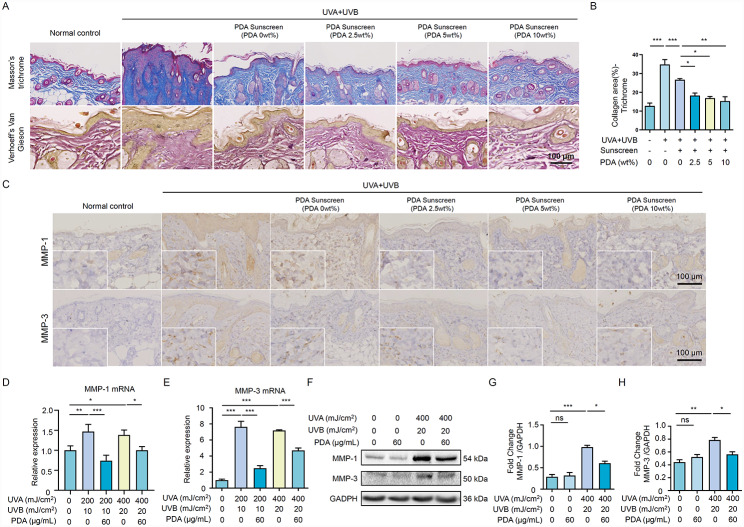
PDA NPs suppress UV-induced collagen degradation and photoaging. (**A**) Representative Masson’s trichrome staining and Verhoeff’s Van Gieson (EVG) staining images of the dorsal skin in Balb/c mice from different groups (n = 4). (**B**) Quantitative analysis of collagen ratio. (**C**) Representative immune histochemistry images of MMP-1 and MMP-3 expression in the dorsal skin of Balb/c mice from different groups (n = 4). (**D, E**) The mRNA expression levels of MMP-1 and MMP-3 of HaCaT cells were detected by qRT-PCR after being pretreated with different concentrations of 60 mg/mL PDA NPs for 24 h, followed by exposure to UVR. The mRNA expression levels of these genes were normalized to β-actin. (**F-H**) The protein level of MMP-1 and MMP-3 of HaCaT cells was determined by western blotting after indicated treatments. GAPDH was detected as a loading control. All data represent the mean ± SD. *p < 0.05, **p < 0.01 and ***p < 0.001

Given that chronic exposure to UVA and UVB rays could accelerate the premature aging process of the skin [25], we assessed the collagen fibers and elastins through Masson’s trichome staining and Verhoeff’s Van Gieson (EVG) staining, respectively. As shown in Fig. 6A, the collagen fibers were pathologically proliferated, meanwhile, the elastins presented fragmented and a disorder in the arrangement. Whereas the application of PDA sunscreen maintained the normal growth metabolism of collagen fibers, as well as the integrity of elastins (Fig. 6A, B). These results reflected PDA sunscreen not just could protect the homeostasis within the epidermis, but also have a promising influence in preserving the healthy state of fibers in the dermis.

The generation of matrix metalloproteinases (MMPs) could mirror premature skin photoaging induced by UV, particularly, MMP-1 and MMP-3 known as collagen destruction enzymes which elevate when inflammation occurs [30]. To determine whether PDA NPs handicap UV-induced collagen degradation, the expression of MMP-1 and MMP-3 were measured in mice after UVR exposure for 9 weeks. As shown in Fig. 6C and B, PDA NPs significantly reduced the expression of MMP-1 and MMP-3 in UV-damaged lesions with a dose-dependent pattern. Whereas, commercial sunscreen only had a marginal effect on MMP-1 and MMP-3 expressions. Furthermore, in vitro cell experiments were carried out to confirm the results observed in the mouse model. qRT-PCR and western blot analysis demonstrated that PDA NPs down-regulated the mRNA and protein levels of MMP-1 and MMP-3 in UV-irradiated HaCaT cells (Fig. 6D-H). Taken together, our results indicated that PDA NPs could inhibit the increased expression of MMP-1 and MMP-3 induced by UVR, consequently preventing collagen degradation and skin photoaging.

## Discussion

While nearly all life forms of the earth live depending on solar UV light, excessive or prolonged exposure to UV radiation can have detrimental effects on living organisms, whose skin would be the first impaired field [1]. For instance, constant exposure to UVR cause both acute damages such as erythema and pigmentation as well as chronic implications including photocarcinogenesis and photoaging [31–33]. Thereupon, we synthesized nanoparticle PDA that mimics melanin. Through multiple performance tests, PDA NPs exhibited high stability, well bio-compatibility, and antioxidant properties. Then, we introduced it to commercial sunscreen to examine its effect on photoprotection. Our results proved that PDA NPs sunscreen performed superior to using commercial sunscreen solely, particularly in scavenging ROS, reducing DNA damage, and confining inflammatory responses, so that to attenuate photodamage. Additionally, PDA NPs sunscreen could inhibit photoaging by preserving the moisture of the skin, protecting the epidermis barrier, and maintaining collagen fibers and elastins organized.

Currently, there is ample evidence validating that the application of sunscreen could help against the harmful effects of UVR [31, 34, 35]. Nevertheless, the poor photostability, low biodegradability, and limited effectiveness of existing commercial sunscreens were unsatisfactory to meet the need of humans for sun protection [9, 36]. Melanin that resided in the epidermis of humans evolved to block deleterious effects induced by solar radiation [37–39]. Inspired by the natural mechanisms to defense UVR, PDA NPs have received great attention in potential applications of photoprotection owing to their melanin-mimicked chemical structure and fundamental light absorption properties [40–42]. Under these conditions, we successfully synthesized PDA NPs with a monodisperse spherical structure. Besides, the incubation of PDA NPs and keratinocytes showed a marginal influence on cell viability, suggesting optimum biocompatibility of PDA NPs. A previous study has reported that PDA NPs were easily endocytosed by human keratinocytes to form a perinuclear cap that protects cell nuclei from UV damage, mimicking the behavior of natural melanosomes and showing minimal cytotoxicity [17]. Beyond this, we further found that PDA NPs were delivered to lysosomes and subsequent accumulation surrounding nuclear to form an artificial cap in keratinocytes, which vividly mimics the biological property of natural melanosomes. Admittedly, the focus of research in this field at this stage is how to prevent UV absorbers from penetrating into the skin. It should be noted that the protective effects of PDA NPs partly function after penetrating into the skin. More notably, PDA NPs have a marginal impact on the viability of keratinocytes, proving to be biocompatible and degradable. These features provide a new dimension for potential application in sunscreen.

Generally, commercial sunscreen composites of either organic filters, inorganic particulate filters, or a combination of them. A recent study has articulated a caution that the mixture of ZnO and small-molecule UV filters poses toxicity in embryonic zebrafish assays [43]. Indeed, the photostability of sunscreens is highly dependent on the mixture of chemicals present. To probe the performance of PDA NPs, we introduced PDA NPs in commercial sunscreen that is based on ZnO. In agreement with Wang et al. [44] who developed three PDA nanocomposite hydrogel sunscreens with excellent UV protection efficiency and high biosafety, our PDA sunscreen could remarkably ameliorate pathological symptoms more superior than using commercial sunscreen solely in the UVR-damaged mice model. Extend to this, we deduced that PDA NPs not only could be photoprotective but also render the system of existing sunscreen more stable. Histologically, we highlighted PDA sunscreen restrains epidermal hyperplasia, deters the infiltration of inflammatory cells, and maintains skin barrier integrality in a concentration-dependent manner.

Keratinocytes, which make up more than 90% of epidermal cells, play a predominant role in photoprotection because they absorb 95% of the UV light that hits the skin [45]. UVR is known to increase oxidative stress and inflammation in the skin, which results in a series of responses that interact together downstream [46]. Previous research has demonstrated the anti-inflammatory capacity of PDA nanoparticles in murine models of both acute peritonitis and acute lung injury (ALI), where diminished ROS generation, reduced proinflammatory cytokines, and attenuated neutrophil infiltration [47]. On the one hand, our findings validated that PDA NPs could drastically inhibit the generation of ROS in keratinocytes stimulated by UVR. On the other hand, we confirmed that PDA NPs reduced the infiltration of neutrophils (indicated by expression of MPO) and the production of UV-related proinflammatory cytokines TNF-α, IL-1β, IL-6, and IL-8.

Given the overproduction of ROS and exacerbated inflammation could trigger or enhance cellular DNA damage which poses a threat to carcinogenesis [46]. It has been reported that PDA administration reversed LPS-elicited DNA damage in hippocampal tissue and medial prefrontal cortex (mPFC) [48]. Our data unveiled that PDA NPs shorten the length of the comet tail and upregulate the expression of the tumor suppressor p53, thereby deterring UV-induced DNA damage *in vivo and in vitro*. This finding highlighted the statement that PDA NPs could form a perinuclear cap around the nucleus of keratinocytes to resist injury under UVR, which is congruent with previous research [17].

So far, UVR is recognized as an essential factor of photoaging, which involves an accumulated synthesis of matrix metalloproteinases (MMPs), reduced abundance of mature collagen, and fragmented elastin [49–51]. MMP-1 and MMP-3 are destructive enzymes that are responsible for the degradation of collagen and elastic fibers, prompting cutaneous photoaging [52, 53]. Mechanically, MMP-1 breaks down collagen initially, followed by MMP-3 [49, 50]. Consistent with previous research [54], our in vivo study found that prolonged UVR exposure led to an aberrantly increased collagen density and poor elasticity in the skin. Notably, we elucidated that PDA sunscreen retains the resilience of skin confronted with UV stress. Collectively, our data indicated that PDA NPs may have a role in remodelling extracellular matrix structures and preventing skin photoaging.

Overall, our results unveiled that PDA sunscreen possesses potent photoprotection ability. Further studies are required to decipher the in-depth mechanism of antioxidant and anti-inflammatory effects of PDA NPs.

## Conclusions

In summary, as new nano-materials mimic natural melanin, PDA NPs provide UV protection not only owing to their absorption of UV but also serve as an antioxidant and anti-inflammation agent. In addition, the sunscreen based on PDA NPs performed superior in reducing the UVR-induced pathological changes (epidermal hyperplasia, transepidermal water loss, and skin barrier disruption). More pronouncedly, PDA sunscreen could inhibit photoaging by maintaining the metabolism of collagen fibers and elastins. Based on our findings, PDA NPs could be harnessed as promising photoprotective agents for the next generation of safe, environmentally friendly, and effective sunscreen.

## Data Availability

All data generated or analysed during this study are included in this published article and its supplementary information files.
